# Inhibition of porcine reproductive and respiratory syndrome virus infection by recombinant adenovirus- and/or exosome-delivered the artificial microRNAs targeting sialoadhesin and CD163 receptors

**DOI:** 10.1186/s12985-014-0225-9

**Published:** 2014-12-19

**Authors:** Li Zhu, Hongqin Song, Xinyu Zhang, Xiaoli Xia, Huaichang Sun

**Affiliations:** College of Veterinary Medicine, Jiangsu Co-Innovation Center for Prevention and Control of Important Animal infectious Diseases and Zoonoses, Yangzhou University, Yangzhou, 225009 China

**Keywords:** Porcine reproductive and respiratory syndrome virus, Receptors, Artificial microRNAs, Anti-viral effect

## Abstract

**Background:**

The current vaccines failed to provide substantial protection against porcine reproductive and respiratory syndrome (PRRS) and the new vaccine development faces great challenges. Sialoadhesin (Sn) and CD163 are the two key receptors for PRRS virus (PRRSV) infection of porcine alveolar macrophages (PAMs), but the artificial microRNA (amiRNA) strategy targeting two viral receptors has not been described.

**Methods:**

The candidate miRNAs targeting Sn or CD163 receptor were predicted using a web-based miRNA design tool and validated by transfection of cells with each amiRNA expression vector plus the reporter vector. The amiRNA-expressing recombinant adenoviruses (rAds) were generated using AdEasy Adenoviral Vector System. The rAd transduction efficiencies for pig cells were measured by flow cytometry and fluorescent microscopy. The expression and exosome-mediated secretion of amiRNAs were detected by RT-PCR. The knock-down of Sn or CD163 receptor by rAd- and/or exosome-delivered amiRNA was detected by quantitative RT-PCR and flow cytometry. The additive anti-PRRSV effect between the two amiRNAs was detected by quantitative RT-PCR and viral titration.

**Results:**

All 18 amiRNAs validated were effective against Sn or CD163 receptor mRNA expression. Two rAds expressing Sn- or CD163-targeted amiRNA were generated for further study. The maximal rAd transduction efficiency was 62% for PAMs at MOI 800 or 100% for PK-15 cells at MOI 100. The sequence-specific amiRNAs were expressed efficiently in and secreted from the rAd-transduced cells via exosomes. The expression of Sn and CD163 receptors was inhibited significantly by rAd transduction and/or amiRNA-containing exosome treatment at mRNA and protein levels. Both PRRSV *ORF7* copy number and viral titer were reduced significantly by transduction of PAMs with the two rAds and/or by treatment with the two amiRNA-containing exosomes. The additive anti-PRRSV effect between the two amiRNAs was relatively long-lasting (96 h) and effective against three different viral strains.

**Conclusion:**

These results suggested that Sn- and CD163-targeted amiRNAs had an additive anti-PRRSV effect against different viral strains. Our findings provide new evidence supporting the hypothesis that exosomes can also serve as an efficient small RNA transfer vehicle for pig cells.

## Background

Porcine reproductive and respiratory syndrome (PRRS) is an economically important swine disease characterized by reproductive failures in sows and respiratory syndromes in pigs of all ages [1]. Since its first outbreak in the United States and Canada in 1987, the disease has been causing heavy economic losses to the pig industry worldwide [2,3]. Although both inactivated and live-attenuated vaccines are available for PRRS control, these vaccines failed to provide sustainable protection against the disease, against heterogeneous viral strains in particular [4]. Porcine reproductive and respiratory syndrome virus (PRRSV) is an enveloped positive–sense RNA virus classified within the *Arteriviridae* family [5,6]. In pigs, the virus targets the cells of monocyte/macrophage lineage [7,8], causing severe cell death, slow and weak antiviral responses, and/or persistent infections. In addition, PRRSV uses additional evasion strategies to escape the host innate and acquired immunity, including interference with antigen presentation, antibody-mediated infection enhancement, reduced cell surface expression of the viral proteins and shielding of the neutralizing epitopes. As a consequence, new PRRS vaccine development faces great challenges since they suffer from the immune evasion strategies of the virus and the highly antigenic heterogeneity of field viral strains [4].

PRRSV enters the target cells by receptor-mediated endocytosis [9]. To date, at least three PRRSV receptors have been identified on porcine alveolar macrophages (PAMs), including heparan sulphate as the general attachment factor, sialoadhesin (Sn or CD169) for the viral binding and internalization, and CD163 for the viral genome release [10]. Previous studies have shown that PRRSV infection of PAMs can be blocked partially by the Sn- or CD163-specific antibody or completely by a combination of two antibodies [11], and that adenoviral (Ad) vector-delivered soluble Sn and CD163 receptors have an additive effect against PRRSV infection [12]. These data suggest that the two viral receptors are the useful targets for designing new strategies for PRRS control.

RNA interference (RNAi) is a post-transcriptional gene silencing mechanism conserved in eukaryotes ranging from worms to humans [13]. Since its discovery in 1994 as an innate antiviral mechanism, RNAi has become a feasible strategy against a variety of viral infections [14]. Two types of small RNAs, namely small interfering RNAs (siRNAs) and microRNAs (miRNAs), are the central players in RNAi process, both of which inhibit gene expression by binding to the target RNA molecules [15]. A recent study has shown that viral vector-expressed artificial miRNAs (amiRNAs) are more effective than the conventional short hairpin RNA (shRNA) strategy [16,17]. Among the viral vectors available, Ads have been used extensively as the gene transfer vectors for gene therapy and vaccine development with several advantages, including efficient gene delivery, transduction of both dividing and non-dividing cells, ease of propagation to high titers, and minimal risk of genomic insertional mutagenesis [18]. In addition, Ad vectors have been used to deliver PRRSV-targeted shRNAs in vitro and in vivo [19]. However, PRRSV targets the cells of monocyte/macrophage which are resistant to rAd transduction due to the lack of high affinity Ad receptor [20]. More recently, it has been shown that the exosomes derived from human and mouse cells can serve as an efficient small RNA transfer vehicle [21]. Therefore, we hypothesized that the co-delivery of Sn- and CD163-targeted amiRNAs by rAds and exosomes could become a novel strategy against PRRSV infection.

To test the above hypothesis, in this study we predicted the candidate miRNAs targeting Sn or CD163 receptor and validated them experimentally using a reporter assay. Two rAds expressing the effective amiRNAs were generated for further study. Cell transduction assays showed that the sequence-specific amiRNAs were expressed efficiently in and secreted from rAd-transduced pig cells via exosomes. In primary PAMs, the expression of two viral receptors was inhibited significantly by transduction with the amiRNA-expressing rAd and/or treatment with the amiRNA-containing exosomes. Furthermore, PRRSV infection of PAMs was inhibited significantly by transduction with the two amiRNA-expressing rAds and/or treatment with the two amiRNA-containing exosomes. These results supported the hypothesis that simultaneous knock-down of Sn and CD163 receptors may become a novel strategy against PRRSV infection. In addition, our findings suggest that exosomes can also serve as an efficient small RNA transfer vehicle for pig cells. To our knowledge, this is the first study to explore an amiRNA strategy against PRRSV infection by targeting the two viral receptors.

## Results

### Prediction and validation of the miRNAs targeting Sn or CD163 receptor

**Figure 1:**
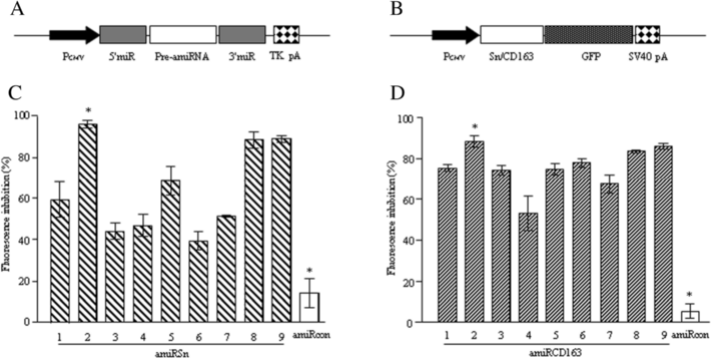
**Validation of Sn and CD163 receptor-targeted amiRNAs. (A)** The schematic structure of amiRNA expression vector. PCMV, immediate early promoter of cytomegalovirus; miR, miR-flanking sequences of mouse BIC non-coding mRNA; Pre-amiRNA, double–stranded oligonucleotide for amiRNA; TK pA, TK gene poly(A) signal of human simplex herpes virus. **(B)** The schematic structure of the reporter vectors for amiRNA validation. PCMV, immediate early promoter of cytomegalovirus; Sn/CD163, porcine Sn or CD163 receptor cDNA; GFP, green fluorescent protein coding sequence; SV40 pA, poly(A) signal of SV40 virus. **(C** or **D)** NIH 3 T3 cells were transfected with different amiRNA expression vector plus the reporter vector, and the GFP-positive cell numbers were measured by flow cytometry 24 h after transfection. The know-down efficiency of each amiRNA was expressed as the percent inhibition of total fluorescence in the cell culture co-transfected with the amiRNA expression vector and the reporter vector.

Computational prediction represents an effective strategy for identification of the candidate siRNAs or miRNAs that can be validated experimentally. We analyzed the first 1751-nt sequence of porcine Sn mRNA and the 1511-nt sequence of porcine CD163 mRNA for candidate miRNAs. Ten top-scoring miRNA sequences for each target were reported and 18 of them (Table 1) were selected for amiRNA vector construction (Figure 1A). To facilitate the candidate miRNA validation, the first 1751-bp sequence of the Sn cDNA or the 1511-bp sequence of the CD163 cDNA was cloned in frame with the green fluorescent protein (GFP) coding sequence in pEGFP-N1 vector to produce the reporter vector pSn-GFP or pCD163-GFP (Figure 1B). NIH 3 T3 cells were co-transfected with each amiRNA expression vector and the reporter vector, and the cell culture was assayed for GFP^+^ cells by flow cytometry. By using pSn-GFP-transfected cells as the reference, transfection with each amiRSn expression vector plus the reporter vector resulted in GFP^+^ cell number reductions ranging from 39.6% to 96.3% (Figure 1C). Similarly, transfection with each amiRCD163 expression vector plus the reporter vector led to GFP^+^ cell number reductions ranging from 53.3% to 88.5% (Figure 1D). The most effective amiRSn-2 and amiRCD163-2, as well as an irrelevant amiRcon (Table 1), were selected for further study.

**Table 1:** **The oligonucleotides used for construction of amiRNA expression vectors in this study**

**Target**	**miRNA**	**Start**	**Double-stranded oligonucleotide sequence (5′→3′)**
Sn	1	161	TGCTG*AGTACCAGATGGCTGTGATGC*GTTTTGGCCACTGACTGACGCATCACACATCTGGTACT
			CCTGAGTACCAGATGTGTGATGCGTCAGTCAGTGGCCAAAACGCATCACAGCCATCTGGTACTC
	2	367	TGCTG*TTTGACATCTGACCAGCGGTT*GTTTTGGCCACTGACTGACAACCGCTGCAGATGTCAAA
			CCTGTTTGACATCTGCAGCGGTTGTCAGTCAGTGGCCAAAACAACCGCTGGTCAGATGTCAAAC
	3	378	TGCTG*ACAACTGTGCCTTTGACATCT*GTTTTGGCCACTGACTGACAGATGTCAGGCACAGTTGT
			CCTGACAACTGTGCCTGACATCTGTCAGTCAGTGGCCAAAACAGATGTCAAAGGCACAGTTGTC
	4	458	TGCTG*AGTTGAAGTCCACCTCCATGC*GTTTTGGCCACTGACTGACGCATGGAGGGACTTCAACT
			CCTGAGTTGAAGTCCCTCCATGCGTCAGTCAGTGGCCAAAACGCATGGAGGTGGACTTCAACTC
	5	461	TGCTG*AGCAGTTGAAGTCCACCTCCA*GTTTTGGCCACTGACTGACTGGAGGTGCTTCAACTGCT
			CCTGAGCAGTTGAAGCACCTCCAGTCAGTCAGTGGCCAAAACTGGAGGTGGACTTCAACTGCT
	6	669	TGCTG*ATCTCCTTCTGCATCCTGCGT*GTTTTGGCCACTGACTGACACGCAGGACAGAAGGAGAT
			CCTGATCTCCTTCTGTCCTGCGTGTCAGTCAGTGGCCAAAACACGCAGGATGCAGAAGGAGATC
	7	1148	TGCTG*AGAAGTAGAAGCCCGAATCCG*GTTTTGGCCACTGACTGACCGGATTCGCTTCTACTTCT
			CCTGAGAAGTAGAAGCGAATCCGGTCAGTCAGTGGCCAAAACCGGATTCGGGCTTCTACTTCTC
	8	1476	TGCTG*TTCCCAAGAGAACTGCTGGCT*GTTTTGGCCACTGACTGACAGCCAGCATCTCTTGGGAA
			CCTGTTCCCAAGAGATGCTGGCTGTCAGTCAGTGGCCAAAACAGCCAGCAGTTCTCTTGGGAAC
	9	1510	TGCTG*TGCATTGGCATGGAAGTCCAG*GTTTTGGCCACTGACTGACCTGGACTTATGCCAATGCA
			CCTGTGCATTGGCATAAGTCCAGGTCAGTCAGTGGCCAAAACCTGGACTTCCATGCCAATGCAC
CD163	1	94	TGCTG*AGAACTAGTGACCAAGCAGGC*GTTTTGGCCACTGACTGACGCCTGCTTTCACTAGTTCT
			CCTGAGAACTAGTGAAAGCAGGCGTCAGTCAGTGGCCAAAACGCCTGCTTGGTCACTAGTTCTC
	2	527	TGCTG*TGAAGTTGTCATCACACACTG*GTTTTGGCCACTGACTGACCAGTGTGTTGACAACTTCA
			CCTGTGAAGTTGTCAACACACTGGTCAGTCAGTGGCCAAAACCAGTGTGTGATGACAACTTCAC
	3	645	TGCTG*CATACAAGATCATCAAACCA*GGTTTTGGCCACTGACTGACCTGGTTTGGATCTTGTATG
			CCTGCATACAAGATCCAAACCAGGTCAGTCAGTGGCCAAAACCTGGTTTGATGATCTTGTATGC
	4	740	TGCTG*TTAAGCAAATCACTCCAGCAT*GTTTTGGCCACTGACTGACATGCTGGAGATTTGCTTAA
			CCTGTTAAGCAAATCTCCAGCATGTCAGTCAGTGGCCAAAACATGCTGGAGTGATTTGCTTAAC
	5	768	TGCTG*ACCACTCTCAGTTTCAGGTCT*GTTTTGGCCACTGACTGACAGACCTGACTGAGAGTGGT
			CCTGACCACTCTCAGTCAGGTCTGTCAGTCAGTGGCCAAAACAGACCTGAAACTGAGAGTGGTC
	6	807	TGCTG*TTCACTTCCAATCTTCCTGAA*GTTTTGGCCACTGACTGACTTCAGGAATTGGAAGTGAA
			CCTGTTCACTTCCAATTCCTGAAGTCAGTCAGTGGCCAAAACTTCAGGAAGATTGGAAGTGAAC
	7	961	TGCTG*AACACTGTCAAGCCAAATGTG*GTTTTGGCCACTGACTGACCACATTTGTTGACAGTGTT
			CCTGAACACTGTCAACAAATGTGGTCAGTCAGTGGCCAAAACCACATTTGGCTTGACAGTGTTC
	8	998	TGCTG*TACACTGCCAGAGAGCAGACT*GTTTTGGCCACTGACTGACAGTCTGCTCTGGCAGTGTA
			CCTGTACACTGCCAGAGCAGACTGTCAGTCAGTGGCCAAAACAGTCTGCTCTCTGGCAGTGTAC
	9	1325	TGCTG*AATTCTTGCAGTCCCAAAGAG*GTTTTGGCCACTGACTGACCTCTTTGGCTGCAAGAATT
			CCTGAATTCTTGCAGCCAAAGAGGTCAGTCAGTGGCCAAAACCTCTTTGGGACTGCAAGAATTC
	10	1486	TGCTG*TAGTGACGAGACAAGCACAGC*GTTTTGGCCACTGACTGACGCTGTGCTTCTCGTCACTA
			CCTGTAGTGACGAGAAGCACAGCGTCAGTCAGTGGCCAAAACGCTGTGCTTGTCTCGTCACTAC
Control	amiRcon		TGCTG*TAGTGACGAGACAAGCACAGC*GTTTTGGCCACTGACTGACGCTGTGCTTCTCGTCACTA
			CCTGTAGTGACGAGAAGCACAGCGTCAGTCAGTGGCCAAAACGCTGTGCTTGTCTCGTCACTAC

The candidate amiRNAs were predicted by following the instructions for the web-based Block-iT^TM^ RNAi designer. The mature amiRNA and forward primer sequences for the detection of amiRNAs in rAd-transduced cells are indicated as italics.

### rAd generation and cell transduction

**Figure 2:**
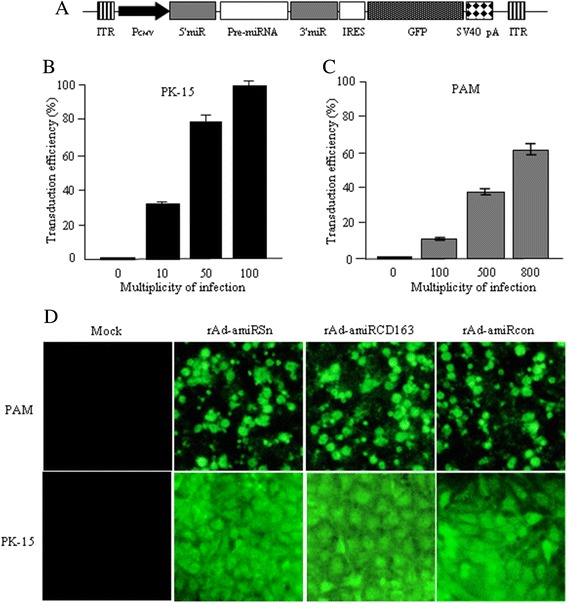
**rAd transduction efficiencies for pig cells. (A)** The schematic structure of Ad vector for amiRNA expression. ITR, inverted terminal repeat of adenovirus; PCMV, immediate early promoter of cytomegalovirus; miR, miR-flanking sequences of mouse BIC non-coding mRNA; Pre-amiRNA, double–stranded oligonucleotide for amiRNA; IRES, internal ribosome entry sequence; GFP, green fluorescent protein coding sequence; SV40 pA, poly(A) signal of SV40 virus. **(B)** PK-15 cells and **(C)** primary PAMs were transduced with different doses of rAd-amiRSn and the GFP-positive cell numbers were measured by flow cytometry 48 h after transduction. **(D)** Primary PAMs and PK-15 cells were mock-transduced or transduced with each rAd and analyzed by fluorescent microscopy (40×) 48 h after transduction.

We subcloned amiRSn-2, amiR163-2 or amiRcon expression cassette into Ad vector pShuttle-IRES-hrGFP, and three rAds, namely rAd-amiRSn, rAd-amiR163 and rAd-amiRcon, were generated by transfecting AAV-293 cells with the rAd vectors (Figure 2A). Primary PAMs and PK-15 cells were transduced with different doses of rAds, and the cell cultures were analyzed for GFP^+^ cells since the GFP reporter gene was included in the rAd vectors. Compared to 100% transduction efficiency for PK-15 cells at MOI 100 (Figure 2B), only 62% transduction efficiency was achieved for PAMs at MOI 800 (Figure 2C), which was not increased further by using higher MOI. The different rAd transduction efficiencies for the two pig cell types were confirmed by fluorescent microscopy (Figure 2D). Therefore, the further experiments were carried out at MOI 800 for PAM transduction and MOI 100 for PK-15 cell transduction.

### The sequence-specific amiRNAs were expressed in and secreted from the rAd-transduced cells

**Figure 3:**
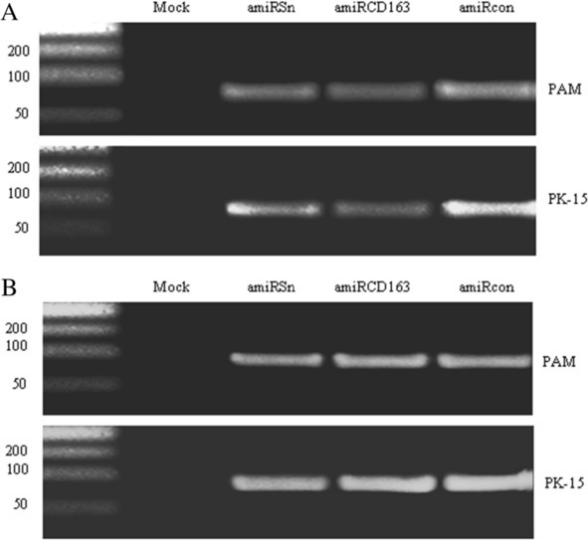
**Detection of amiRNA expression in the rAd-transduced cells and purified exosomes by RT-PCR. (A)** Primary PAMs and PK-15 cells were mock-transduced or transduced with each rAd, and the total RNA was extracted for sequence-specific amiRNA detection 48 h after transduction. **(B)** The exosomes were purified from the cell culture medium of each rAd-transduced cells and the total RNA was extracted for sequence-specific amiRNA detection 48 h after transduction.

To investigate whether the amiRNAs were expressed in rAd-transduced cells, primary PAMs or PK-15 cells were transduced with rAd-amiRSn, rAd-amiRCD163 or rAd-amiRcon, and the total RNA was extracted 48 h after transduction. At the same time, the exosomes were purified from cell culture medium and the total RNA was extracted for sequence-specific amiRNA detection. The expected 80-nt amiRNAs were detected in all of the three rAd-transduced cells (Figure 3A) and their exosomes (Figure 3B), but not in the mock-transduced cells and their exosomes.

### The rAd- and exosome-delivered amiRNA had an additive effect against the target gene expression

**Figure 4:**
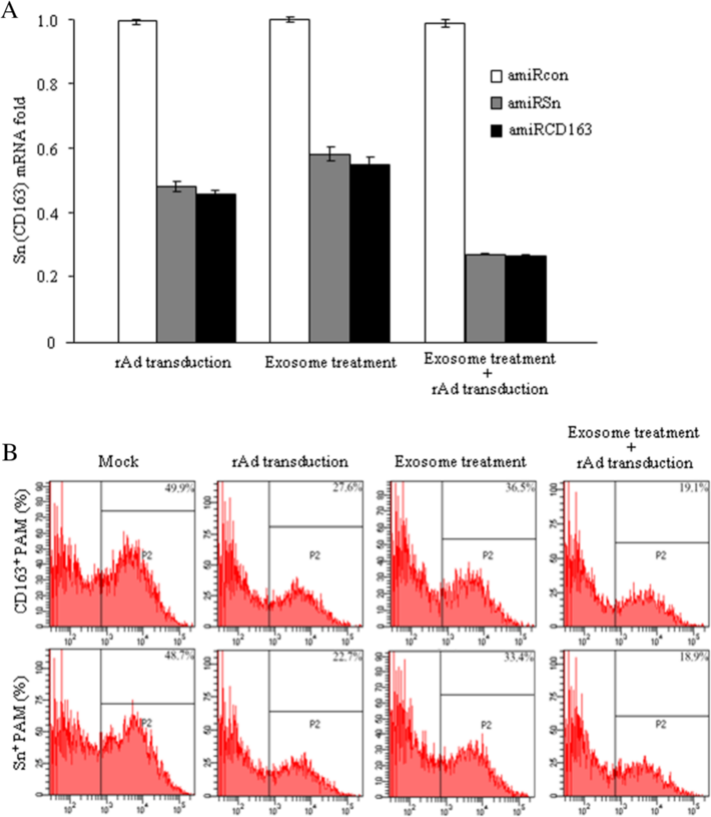
**Knock-down of Sn or CD163 receptor by the rAd- and/or exosome-delivered amiRNA. (A)** Primary PAMs were transduced with each rAd and/or incubated with each amiRNA-containing exosomes derived from the rAd-transduced PK-15 cells. At 48 h after incubation, the total RNA was extracted for Sn or CD163 mRNA detection by real-time quantitative RT-PCR. **(B)** The rAd-transduced and/or exosome-incubated cells were stained with Sn- or CD163-specific antibody and analyzed by flow cytometry.

The sufficient knock-down of Sn and CD163 receptors is the precondition for the objective evaluation of anti-PRRSV effects of the receptor-targeted amiRNAs. We compared the knock-down efficiencies of the two viral receptors using three different strategies: rAd transduction, exosome treatment and rAd transduction plus exosome treatment. First, primary PAMs were transduced with rAd-amiRSn, rAd-amiR163 or rAd-amiRcon. After incubation for 48 h, the total RNA was extracted for mRNA detection by quantitative RT-PCR and the cell culture was analyzed for Sn^+^ or CD163^+^ cell number by flow cytometry. Compared to that in rAd-amiRcon-transduced cell cultures, Sn or CD163 receptor mRNA expression in the rAd-amiRSn- or rAd-amiRCD163-transduced cells was decreased by 0.51 or 0.54 fold (Figure 4A), while Sn^+^ and CD163^+^ cell numbers were decreased from 48.7% to 22.7% and from 49.9% to 27.6%, respectively (Figure 4B). Next, primary PAMs were incubated with the exosomes (0.5 mg protein/ml) derived from rAd-amiRSn- or rAd-amiRCD163-transduced PK-15 cells. At 48 h after incubation, the total RNA was extracted for quantitative RT-PCR and cell cultures were harvested for flow cytometry analysis as described. Compared to that in the two control groups, Sn or CD163 mRNA expression in the amiRNA-containing exosome-incubated cells was decreased by 0.41 or 0.46 fold (Figure 4A), while Sn^+^ and CD163^+^ cell numbers were decreased to 33.4% and 36.5%, respectively (Figure 4B). Finally, primary PAMs were transduced first with each rAd, incubated for 48 h with each amiRNA-containing exosomes, the total RNA was extracted for quantitative RT-PCR and the cell cultures were harvested for flow cytometry analysis as described. Compared to that in the two control groups, Sn or CD163 mRNA expression was decreased by 0.73 or 0.73 fold (Figure 4A), while Sn^+^ and CD163^+^ cell numbers were decreased to 18.9% and 19.1%, respectively (Figure 4B).

### Sn and CD163 receptor-targeted amiRNAs had an additive effect against PRRSV infection

**Figure 5:**
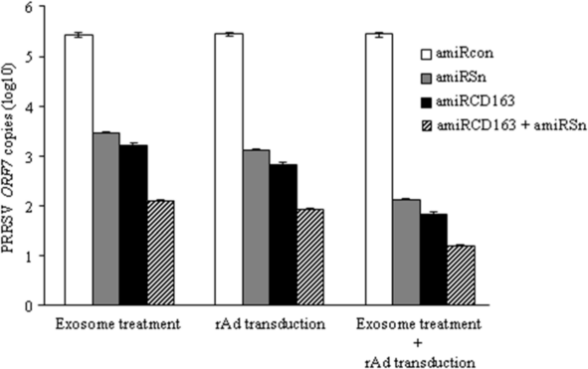
**Detection of PRRSV** ***ORF7*** **copy numbers in rAd-transduced and/or exosome-incubated PAMs.** Primary PAMs were transduced with single or double rAds and/or incubated for 48 h with single or double amiRNA-containing exosomes. The cells were infected with PRRSV strain VR-2332 and the total RNA was extracted 24 h post infection for *ORF7* copy number detection by real time quantitative RT-PCR.

We used two different quantitative assays to evaluate the additive anti-PRRSV effect between Sn and CD163 receptor-targeted amiRNAs: quantitative RT-PCR and viral titration. For the quantitative RT-PCR, primary PAMs were transduced with rAd-amiRcon, rAd-amiRSn and/or rAd-amiRCD163 as described, and infected with PRRSV strain VR2332 (MOI 0.2) 48 h after transduction. At 24 h post infection, the total RNA was extracted for quantitative RT-PCR using PRRSV *ORF7*-specific primers (Table 2). Compared to that (5.4 log10) in rAd-amiRcon-transduced cells, the *ORF7* copy number in rAd-amiRSn and/or rAd-amiRCD163 transduced cells was decreased by 3.5, 2.3 or 2.6 log10 (Figure 5). Next, primary PAMs were incubated with the exosomes derived from rAd-amiRSn- and/or rAd-amiRCD163-transduced PK-15 cells, infected with PRRSV and the total RNA was extracted for quantitative RT-PCR as described. Compared to that (5.4 log10) in the control group, the *ORF7* copy number in amiRSn- and/or amiRCD163-containing exosome-incubated cells was decreased by 3.3, 2.0 or 2.2 log10 (Figure 5). Finally, primary PAMs were transduced with different rAds, incubated with different amiRNA-containing exosomes, infected with PRRSV and total RNA was extracted for RT-PCR as described. Compared to that (5.4 log10) in the control group, the *ORF7* copy number in double rAd-transduced and double amiRNA-containing exosome-incubated cells was decreased by 4.2 log10, while the *ORF7* copy number in single rAd-transduced and single amiRNA-containing exosome-incubated cells was decreased by 3.3 or 3.6 log10 (Figure 5).

**Table 2:** **The primers used for PCR or real-time quantitative PCR in this study**

**Gene**	**Primer pair**	**Sequence (5′→3′)**	**Amplicon (bp)**
Sn	For1	CTAGATCTATGGACTTCCTGCTCCTGCT	1751
	Rev1	CTGTCGACCTGGTGCTGTGGCTGTTCTG	
	For2	AGCAGCCGAACGCAGGAT	201
	Rev2	TTCTGGTCTTTGAGCTTCGTCC	
CD163	For1	CTCTCGAGATGGTGCTACTTGAAGACTC	1511
	Rev1	CTGGATCCTCCAGAGAGAAGTCAGAATC	
	For2	ATTCATCATCCTCGGACCCAT	110
	Rev2	CCCAGCACAACGACCACCT	
PRRSV	For	CCAGCCAGTCAATCARCT	208
ORF7	Rev	GCGAATCAGGCGCACWGTATG	
GAPDH	For	ACACTCACTCTTCTACCTTTG	90
	Rev	CAAATTCATTGTCGTACCAG	

**Figure 6:**
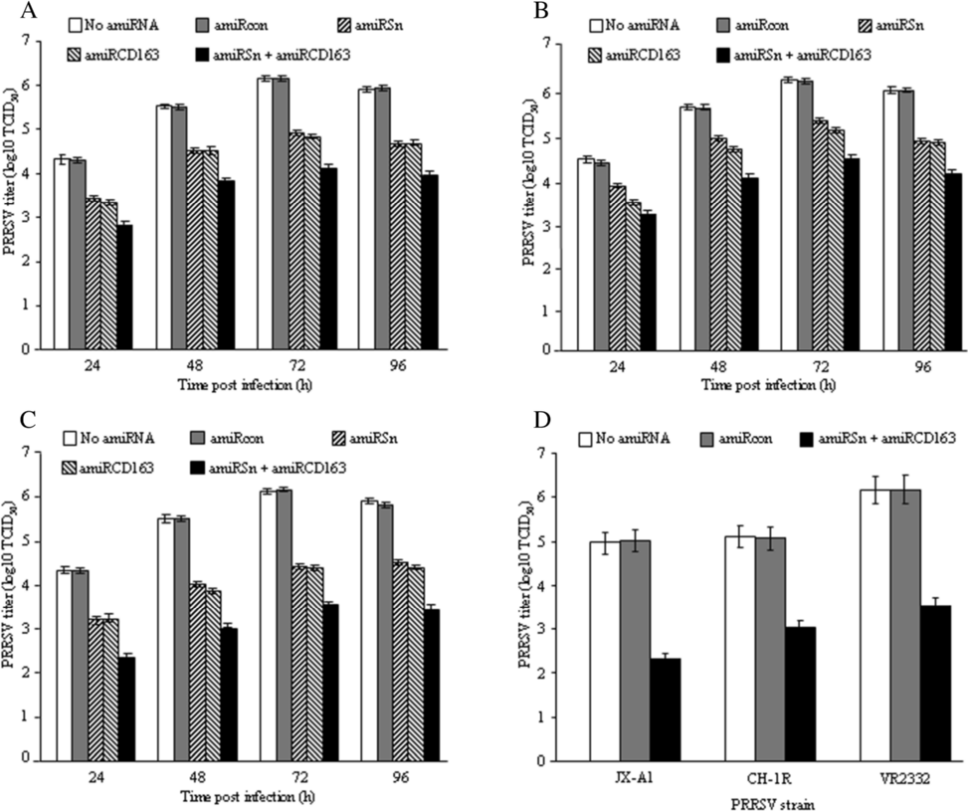
**Titration of PRRSV titers in rAd-transduced and/or exosome-incubated PAMs. (A)** Primary PAMs were transduced with different rAds and infected with PRRSV strain VR2332 48 h after transduction. At different time points after infection, the cells were harvested for PRRSV titration on MARC-145 cells. **(B)** Primary PAMs were incubated for 48 h with different amiRNA-containing exosomes and then infected with PRRSV strain VR2332 before PRRSV infection and titration. **(C)** Primary PAMs were transduced with different rAds and then incubated for 48 h with different amiRNA-containing exosomes before PRRSV infection and titration. **(D)** Primary PAMs were transduced with different rAds and then incubated for 48 h with different amiRNA-containing exosomes. The cells were infected with three different PRRSV strains and harvested for PRRSV titration 72 h after infection.

For the viral titration assay, primary PAMs were transduced with different rAds and infected with PRRSV strain VR2332 as described. At different time points post infection, the cells were harvested for PRRSV titration on MARC-145 cells. Compared to that (4.3 log10 TCID_50_) in the mock- or rAd-amiRcon-transduced cells, the PRRSV titer in rAd-amiRSn- and/or rAd-amiRCD163-transduced cells was decreased by 1.5, 0.9 or 1.0 log10 at 24 h post infection (Figure 6A). Next, primary PAMs were incubated with amiRSn- and/or amiRCD163-containing exosomes, infected with PRRSV and harvested for PRRSV titration as described. Compared to that (4.3 or 4.2 log10 TCID_50_) in the mock- or amiRcon-containing exosome-treated cells, PRRSV titer in amiRSn- and/or amiRCD163-containing exosome-incubated cells was decreased by 1.2, 0.6 or 1.0 log10 (Figure 6B). Then, primary PAMs were transduced with different rAds, incubated with different amiRNA-containing exosomes, infected with PRRSV and harvested for PRRSV titration as described. Compared to that (4.3 or 4.4 log10 TCID_50_) in the two control groups, the PRRSV titer in double rAd-transduced and double amiRNA-containing exosome-incubated cells was decreased by 2.0 log10, while the viral titer in single rAd-transduced and single amiRNA-containing exosome-incubated cells was decreased by 1.1 log10 (Figure 6C). The similar additive anti-PRRSV effect between the two amiRNAs lasted for at least 96 h (Figure 6A,B or C). Finally, primary PAMs were transduced with different rAds, incubated with different amiRNA-containing exosomes and infected with PRRSV strain JX-A1, CH-1R or VR2332 as described. At 72 h post infection, the infected cells were harvested for PRRSV titration. Compared to that (4.9-6.2 log10 TCID_50_) in the two control groups, the PRRSV titers in three viral strain-infected cells were decreased by 2.7, 2.1 and 2.6 log10, respectively (Figure 6D).

## Discussion

As a natural antiviral mechanism, RNAi has become a feasible strategy against a variety of viral infections [13], but its in vivo use is limited by the low efficiency of small RNA delivery. Such drawbacks can be alleviated by using viral vectors for small RNA delivery. Among the viral vectors available, Ads have several advantages and thus have been used extensively as the gene transfer vectors for gene therapy and vaccine development [18]. Furthermore, Ads have been shown to an efficient shRNA delivery vehicle for pig cells [19]. Therefore, in this study we used rAd vectors as the amiRNA delivery vehicle. Cell transduction experiments showed that the human Ad5-based vectors could not only transduce pig cells (Figure 2), but also express the encoded amiRNAs efficiently (Figure 3). Unlike pig kidney PK-15 cells, however, primary PAMs were resistant to rAd transduction and only a maximal transduction efficiency of about 60% was achievable by using very high doses (MOI ≥ 800) of rAd vectors (Figure 2). This could be explained by the lack of high affinity Ad receptor on primary PAMs, which has been reported for the primary macrophages in other species [20].

As a natural mechanism in animal cells, the exosomes derived from human and mouse cells can serve as an efficient vehicle for small RNA transfer [21]. In the light of resistance of PAMs to rAd transduction, this warranted us to explore the feasibility of pig cell-derived exosomes as a small RNA delivery vehicle, which has not been reported in livestock such as pigs. Our RT-PCR assay showed that the sequence-specific amiRNAs were easily detected in the exosomes purified from the cell culture medium of rAd-transduced pig cells (Figure 3B), indicating that the viral vector-encoded amiRNAs were secreted from the pig cells via exosomes. Furthermore, the sequence-specific amiRNAs were detected in the exosome-incubated PAMs (data not shown) and showed significant inhibitory effects against PRRSV infection (Figures 5 and 6). These results suggested that exosomes could also serve as an efficient small RNA transfer vehicle for pig cells.

PRRSV uses at least three receptors to enter PAMs, among which Sn and CD163 play essential but different roles [10]. It has been shown that PRRSV infection of PAMs can be blocked partially by Sn- or CD163-specific antibody, or completely by a combination of two antibodies [11]. Our previous study has also shown that the soluble Sn and CD163 receptors have an additive effect against PRRSV infection [12]. These indicate that the simultaneous knock-down of two viral receptors is required to achieve a significant antiviral effect. In the light of resistance of PAMs to rAd transduction, we compared Sn and CD163 receptor knock-down efficiencies of three different strategies: rAd transduction, exosome treatment and rAd transduction plus exosome treatment. Both quantitative RT-PCR and flow cytometry analyses showed that, compared to the lower knocking-down efficiency of Sn or CD163 receptor by rAd transduction or exosome treatment, the rAd transduction plus exosome treatment resulted in significantly more knock-down of the two viral receptors at both mRNA and protein levels (Figure 4). These results suggested that exosomes could assist rAds to deliver small RNAs into pig cells, which was particularly important for the delivery of amiRNAs into PAMs that were resistant to rAd transduction.

Similarly, we used the three different strategies to investigate the additive anti-PRRSV effect between Sn and CD163 receptor-targeted amiRNAs. Quantitative RT-PCR showed that the simultaneous knock-down of two viral receptors resulted in more reductions in PRRSV *ORF7* copy number than the single receptor knock-down (Figure 5). In addition, much more reduction in the *ORF7* copy number was achieved by using rAd transduction plus exosome treatment (Figure 5). The similar results were obtained from viral titration assays (Figure 6,B and C). The additive anti-PRRSV effect between the two viral receptor-targeted amiRNAs was relatively long-lasting (96 h) and effective against all three different viral strains tested (Figure 6D). However, the complete inhibition was not achieved by using three different strategies and very high rAd doses (MOI ≥ 800) for cell transduction. This could be explained by the following reasons: first, Sn and/or CD163 receptor-targeted amiRNAs delivered by three different strategies were insufficient for the complete know-down of two viral receptors (Figure 4) due to the inherent incomplete knock-down of RNAi strategy [22], and/or the resistance of primary PAMs to rAd transduction [20]. Second, the turnover of Sn and/or CD163 receptor was longer than that we tested (48 h) and thus the certain amounts of pre-expressed receptors were present on the rAd-transduced and/or exosome-incubated PAMs. Finally, there were alternative cellular factor (s) may be involved in PRRSV infection of primary PAMs. Among these, CD151 may be an important one since it plays an important role in PRRSV infection of MARC-145 cells and is expressed on primary PAMs [23,24]. These warrant us to refine the amiRNA strategy by targeting more cellular factors and/or using more efficient amiRNA transfer vectors such as recombinant lentiviruses.

Both Sn and CD163 receptors are macrophage-restricted cell surface molecules that are conserved across mammals. Among these, Sn receptor is a member of the sialic acid-binding IgG-like lectin family of proteins which contributes to sialylated pathogen uptake, antigen presentation and lymphocyte proliferation [25], while CD163 receptor is a critical for the efficient extracellular hemoglobin clearance during hemolysis [26]. Therefore, whether the knock-down of two receptors could influence the viability or compromise the cell functions of PAMs should be considered. In the light of the fact that expression of the two receptors are regulated by several factors such as glucocorticoids and IL-10, and that the expression levels vary significantly under different conditions [15,26], we speculated that the two genes were not vital for the cell viability. This speculation was supported by an additional fact that the viability of PAM cell lines is not compromised by the loss of Sn and/or CD163 expression [27]. In any case, more studies are certainly needed to address the safety of our amiRNA strategy before its in vivo use.

In summary, in this study we generated two rAds expressing Sn or CD163 receptor-targeted amiRNA and investigated their anti-PRRSV effect using different strategies. The Ad vectors could not only transduce different pig cells, but also express the vector-encoded amiRNAs. The vector-expressed amiRNAs were secreted from rAd-transduced cells via exosomes not only, but taken up by other pig cells as well. A significant additive anti-PRRSV effect between the two amiRNAs was demonstrated by transduction of PAMs with the two rAds or by incubation with the two amiRNA-containing exosomes, which was enhanced further by co-transduction with the two rAds plus co-incubation with the two amiRNA-containing exosomes. According to our knowledge, this is the first study to explore an amiRNA strategy against PRRSV infection by targeting the two viral receptors. In addition, we provided new evidence that the exosomes could also serve as an efficient small RNA deliver vehicle for pig cells. Although the complete anti-PRRSV effect has not yet been demonstrated, this study may facilitate further works on development of more efficient strategies against PRRS.

## Conclusion

This study demonstrated that Sn and CD163 receptor-targeted amiRNAs had an additive anti-PRRSV effect against different viral strains. In addition, we provided new evidence that the exosomes could serve as an efficient small RNA deliver vehicle for pig cells.

## Methods

### Cells, viruses and reagents

Mouse fibroblast cell line L-929 (ATCC CCL-1) was grown in Dulbecco’s Modified Eagle’s Medium (DMEM)/Nutrient Mixture F-12 (Gibco, USA) supplemented with 10% fetal bovine serum (FBS) and 1% non-essential amino acids. Pig kidney cell line PK-15 (ATCC CCL-33), mouse embryo fibroblast cell line NIH 3 T3 (ATCC CRL-1658), AAV-293 cells (Stratagene, USA) and African green monkey kidney cell line MARC-145 cells (ATCC CRL-12231) were grown in DMEM supplemented with 10% FBS and 1% non-essential amino acids. Primary PAMs were prepared from 6-week-old Lancrace pigs as described previously [7] and grown in DMEM/F-12 supplemented with 10% FBS, 1% non-essential amino acids and 20% L-929-conditioned medium. PRRSV strain VR-2332 (ATCC VR2332) is a prototype strain of North American genotype [28]. PRRSV strain CH-1R is an attenuated vaccine strain derived from the traditional Chinese strain CH-1a [29]. PRRSV strain JX-A1 is a highly pathogenic Chinese strain [30]. The three PRRSV strains were propagated and titrated on MARC-145 cells.

### miRNA prediction and double-strand oligonucleotide prepration

The first 1751-nt sequence of porcine Sn mRNA (GenBank: AF505985) and the 1511-nt sequence of porcine CD163 mRNA (GenBank: NM_213976) were predicted using the web-based BLOCK-iT™ RNAi designer (Invitrogen, USA). Among 10 top-scoring sequences reported for each target, 18 of them were selected for miR RNAi design according to their higher knock-down probabilities (star rankings). The top- and bottom-strand oligonucleotides (Table 1) were synthesized, each pair of them (5 μl, 200 μM) was denatured at 94°C for 5 min and slowly annealed at room temperature to form double-strand oligonucleotide.

### Vector construction

For construction of the amiRNA expression vectors, each double-strand oligonucleotide was cloned at the *Bbs*I site of amiRNA expression vector pcDNA-miR [31], in which the amiRNA expression cassette consists of the 5′- and 3′-miR flanking sequences of mouse BIC non-coding mRNA, followed by the poly(A) signal of human herpesvirus TK gene (Figure 1A). The recombinant vectors, namely pcDNA-miRSn (1–9), pcDNA-miRCD163 (1–9) or control pcDNA-miRcon, were used for amiRNA validation.

For construction of the reporter vectors, the cellular RNA was extracted from primary PAMs using RNAiso Plus (KaTaRa, Dalian, China) according to the manufacturer’s instructions. The reverse transcription (20 μl) was performed using RevertAid™ Reverse Transcriptase (Fermentas, USA) by following the manufacturer’s protocol. The PCR (50 μl) was performed using 5 μl of RT product and LA Taq DNA Polymerase (KaTaRa, Dalian, China) according to the manufacturer’s manual. The first 1751-bp Sn cDNA segment was amplified using primers Sn For1 and Sn Rev1 (Table 2). The PCR was carried out at 94°C for 5 min; 30 cycles of 94°C for 45 sec, 60°C for 45 sec and 72°C for 2 min; and a final extension at 72°C for 10 min. The first 1511-bp CD163 cDNA segment was amplified using primers CD163 For1 and CD163 Rev1 (Table 2). The PCR was carried out at 94°C for 5 min; 30 cycles of 94°C for 45 sec, 50°C for 45 sec and 72°C for 1.5 min; and a final extension at 72°C for 10 min. Each PCR product was cloned into pMD18-T vector for sequencing and then at the *Xho*I/*Bam*HI site of pEGP-N1 vector (Invitrogen, USA) to produce reporter vector pSn-EGFP or pCD163-EGFP (Figure 1B). The expression of two GFP fusions was checked by transfection of NIH 3 T3 cells and fluorescent microscopy.

For construction of the rAd vectors, the amiRSn-2, amiRCD163-2 or amiRcon expression cassette was excised from the amiRNA expression vector by restriction digestion with *Aat*II and *Pvu*I, and cloned into pShuttle-IRES-hrGFP (Stratagene, USA) vector after linerization with the same enzymes. The pShuttle-IRES-hrGFP vector is rendered replication defective by deletion of the E1 and E3 genes [32], and by inclusion of humanized Renilla reniformis GFP (hrGFP) sequence after the internal ribosome entry site (IRES) for follow-up purpose. The resultant rAd vector was called pShuttle-amiRSn-IRES-hrGFP, pShuttle-amiRCD163-IRES-hrGFP or pShuttle- amiRcon-IRES-hrGFP (Figure 2A).

### Candidate amiRNA validation

NIH 3 T3 cells were seeded in triplicates at 5 × 10^5^/well on 24-well plates and grown to 70% confluent growth. The cells were transfected with pcDNA-amiRSn (1–9), pcDNA-amiRCD163 (1–9) or pcDNA-amiRcon (2 μg/well) plus pSn-EGFP or pCD163-EGFP (0.4 μg/well). The transfection was performed using Lipofectamine™ 2000 (Invitrogen, USA) according to the manufacturer’s manual. At 24 h after transfection, the cells were trypsinized, washed three times with PBS, diluted to 10^6^ cells/ml and analyzed (10^4^ cells) for total fluorescence and cell numbers on BD FACSAia III (BD, USA). The effectiveness of each amiRNA was expressed as the percent inhibition of Sn-GFP or CD163-GFP fusion expression using pSn-GFP- or pCD163-GFP-transfected as the reference.

### rAd preparation and cell transduction

The three rAds, namely rAd-amiRSn, rAd-amiRCD163 and rAd-amiRcon, were generated by transfecting AAV-293 cells (Stratagene, USA) with the rAd vectors according to the instruction manual for AdEasy™ Adenoviral Vector System (Agilent Technologies, USA). The rAds were amplified on AAV-293 cells, purified using ViraBind™ Adenovirus Miniprep Kit (CELL BIOLABS, USA) by following the manufacturer’s protocol, and titrated on AAV-293 cells as fluorescent formation units (FFU)/ml. For transduction of PK-15 cells, the cells were seeded in triplicates at 5 × 10^4^ cells/well on 24-well plates, and grown to 80% confluent growth. After two time wash with PBS, the cells were transduced (37°C for 2 h) with different doses (MOI) of rAds. The transduction of primary PAMs was carried out as previously described [33]. Briefly, the cells were cultured for 7 days in the growth medium conditioned with 20% of L-929 cell culture medium. The cells were trypsinized, diluted to 10^5^cells/ml with the same medium, mixed with different doses of rAds and centrifuged at 2000 × g for 1 h at 37°C. After wash again with PBS, the cells were grown for additional 48 h in the fresh medium and assayed for GFP-positive cells on BD FACSAia III or by fluorescent microscopy.

### Exosome purification

Exosome purification was performed as previously described [34]. Briefly, primary PAMs and PK-15 cells were seeded in T75 flasks and grown to 70% confluent growth in the medium supplemented with 10% exosome-depleted FBS. The cells were transduced with each rAd (MOI 800 for primary PAMs or 100 for PK-15 cells) and incubated for additional 48 h. The cell medium was collected and centrifuged at 4°C, 300 g for 10 min, 1000 g for 30 min, 10,000 g for 45 min to remove the cell debris. After filtration through a 0.22-μm filter membrane, the exosomes were precipitated by centrifugation at 120,000 g for 70 min and suspend in PBS for immediate use or stored at −80°C for later use.

### Poly(A)-tailed RT-PCR

The poly(A)-tailed RT-PCR for amiRNA detection was performed using One Step Prime Script miRNA cDNA Synthesis Kit (KaTaRa, Dalian China) by following the manufacturer’s protocol. Briefly, the total RNA was extracted from rAd-transduced cells or purified exosomes for cDNA synthesis. Each amiRNA cDNA was amplified using the sequence-specific forward primer (Table 1) and the general reverse primer in the cDNA synthesis kit. The PCR was carried out at 95°C for 30 sec, 35 cycles of 95°C for 10 sec, 58°C for 30 sec, 72°C for 10 sec, and a final extension at 72°C for 5 min. The PCR products (8 μl) were analyzed by electrophoresis on a 3% agarose gel.

### Quantitative RT-PCR

The real-time quantitative RT-PCR for Sn and CD163 receptor mRNA detection was performed as described previously [35]. Briefly, primary PAMs were transduced in triplicates with each rAd and incubated in the medium with or without the exosomes (0.5 mg protein/ml) purified from the rAd-transduced PK-15 cells. At 48 h after incubation, the total RNA was extracted for reverse transcription using RevertAid^TM^ Reverse Transcriptase (Fermentas, USA). The Sn and CD163 receptor mRNA expression was analyzed by SYBR green-based real-time quantitative PCR using Roche LightCycler Nano system. The mRNA copy number was normalized by comparing to house-keeping GAPDH copy number. The primer sequences are listed in Table 2.

The real-time quantitative RT-PCR for PRRSV *ORF7* copy number detection was performed as previously described [36]. Briefly, primary PAMs were transduced in triplicates with different rAds and/or incubated in the growth medium with or without the exosomes derived from rAd-transduced cells as described. At 48 h after incubation, the cells were infected with PRRSV (MOI 0.2), incubated for additional 24 h and total RNA was extracted for reverse transcription and real-time quantitative PCR as described. The standard curve was generated by comparing to PRRSV *ORF7*-containing plasmid [36]. The primer sequences are listed in Table 2.

### Flow cytometry analysis for detection of Sn and CD163 receptor knock-down

The flow cytometry for detection of Sn and CD163 receptor knock-down was performed as previously described [37]. Briefly, primary PAMs were transduced in triplicates with different rAds and/or incubated for 48 h with or without the exosomes derived from rAd-transduced cells. The cells were stained with Sn- or CD163-specific antibody and analyzed by flow cytometry as described.

### PRRSV infection and titration

Primary PAMs were seeded at 2 × 10^5^/ml on 24-well plates and grown overnight in the medium supplemented with 10% exosome-depleted FBS. The cells were mock-transduced in triplicates or transduced with different rAds and/or incubated for 48 h with or without the exosomes (0.5 mg protein/ml) purified from different rAd-transduced PK-15 cells. The cells were infected with PRRSV strain VR-2332, CH-1R or JX-A1 (MOI 0.2) and harvested at different time points for PRRSV titration on MARC-145 cells as previously described [38].

## Abbreviations

PRRS: Porcine reproductive and respiratory syndrome
PRRSV: Porcine reproductive and respiratory syndrome virus
siRNA: Small interfering RNA
miRNA: microRNA
FBS: Fetal bovine serum
DMEM: Dulbecco’s modified eagle’s medium
PCR: Polymerase chain reaction
RT-PCR: Reverse transcriptase PCR
MOI: Multiplicity of infection
TCID_50_: 50% tissue culture infective dose
